# Highly expressed genes evolve under strong epistasis from a proteome-wide scan in *E. coli*

**DOI:** 10.1038/s41598-017-16030-z

**Published:** 2017-11-20

**Authors:** Pouria Dasmeh, Éric Girard, Adrian W. R. Serohijos

**Affiliations:** 1 Departement de Biochimie, Université de Montréal, 2900 Édouard-Montpetit, Montréal, Québec, H3T 1J4 Canada; 2 Centre Robert Cedergren en Bioinformatique et Génomique, Université de Montréal, 2900 Édouard-Montpetit, Montréal, Québec, H3T 1J4 Canada

## Abstract

Epistasis or the non-additivity of mutational effects is a major force in protein evolution, but it has not been systematically quantified at the level of a proteome. Here, we estimated the extent of epistasis for 2,382 genes in *E. coli* using several hundreds of orthologs for each gene within the class *Gammaproteobacteria*. We found that the average epistasis is ~41% across genes in the proteome and that epistasis is stronger among highly expressed genes. This trend is quantitatively explained by the prevailing model of sequence evolution based on minimizing the fitness cost of protein unfolding and aggregation. The genes with the highest epistasis are also functionally involved in the maintenance of proteostasis, translation and central metabolism. In contrast, genes evolving with low epistasis mainly encode for membrane proteins and are involved in transport activity. Our results highlight the coupling between selection and epistasis in the long-term evolution of a proteome.

## Introduction

Resolving the link between genotype and phenotype or the fitness landscape is a central goal in molecular biology and evolution. Knowledge of the structure of the fitness landscape will lead to a better understanding of the evolutionary origin of natural proteins and to solutions to practical evolutionary problems, from rational design of enzymes to the development of new antibiotics^1^. The fitness landscape is complex and a consequence of this complexity is *epistasis* or the dependence of mutational effects on genetic background^2^. The presence of epistasis implies that the effects of multiple mutations are non-additive and that their order of fixation matters. Indeed, epistasis directly affects the potential pathways to explore the fitness landscape^2^. Despite the many experimental and theoretical studies on detecting and elucidating its role in molecular evolution^3,4^, none has investigated the strength of epistasis at a proteome-wide level. Such an analysis can determine correlations between epistasis and genomics properties that could hint at a universal mechanism, if any, for epistasis in proteome evolution. Additionally, a mechanistic understanding of epistasis has practical applications; as yet, it is rarely accounted for in the molecular evolution toolboxes for quantifying from genomic sequences the strength of multiple evolutionary forces—mutation, drift and selection^4,5^.

Epistasis, or the non-additivity of mutational effects, has a direct role on the rate of protein evolution because this implies that the genetic background can attenuate the effect of the mutation and hence affect its likelihood of fixation. Nonetheless, this relationship between epistasis and rate of evolution is complex because of confounding factors that also affect the rate of evolution, most important of which is selection. To estimate the epistasis experienced by genes in long-term evolution, one approach is to compare two rates of amino acid substitutions^6^. These two rates are the average pairwise substitution rate *R*_dN/dS_, which is background-dependent, and the rate of mutational usage *R*_*u*_, which is background independent. Both rates are calculated from a multiple sequence alignment (MSA) of orthologs. Specifically, the extent of epistasis is quantified as

1 $$\varepsilon =1-\frac{{R}_{dN/dS}}{{R}_{u}}$$

*R*_dN/dS_ is the average d*N*/d*S* (the ratio of non-synonymous substitution rate *dN* and synonymous substitution rate *dS*) for all pairs of orthologues in an MSA. *R*_dN/dS_ is calculated over the entire length of the gene, thus it reflects the co-evolution between sites. This also implies that *R*_dN/dS_ accounts for the background- and lineage-specificity of amino acid substitutions. The second rate,

$${R}_{u}=(\frac{1}{L}){\sum }_{i}^{L}\frac{({u}_{i}-1)}{19}$$

where *u* is the mutational usage and is the number of unique amino acids in each site in an MSA. *L* is the length of the protein. *R*_*u*_ represents the ratio between observed accessible amino acid substitutions in a site, (*u*−1), and all possible amino acid substitution assuming no selection, that is, (20−1) = 19. Unlike *R*_dN/dS_, *R*_u_ simply counts the number of unique amino acids per site, thus it does not reflect the co-evolution between sites in the protein. Therefore, *R*_*u*_ is independent of background and lineage. When all mutations are neutral, both *R*_*u*_ and *R*_*dN/dS*_ are equal to 1. When *random* mutations are not neutral, such as in proteins where they are predominantly destabilizing and deleterious^7^, purifying selection will lead to *R*_*u*_ and *R*_*dN/dS*_ less than 1. However, the presence of epistasis implies that genetic background further screens substitutions, thus the background dependent *R*_*dN/dS*_ is slower than the background independent *R*_*u*_. The expression for epistasis (Eq. 1) estimates how much epistasis or the background specificity of mutational effects slows down the rate of protein evolution. Kondrashov and coworkers^6^ applied this method to estimate epistasis in the long-term evolution of 16 mammalian proteins and found epistasis to vary from ~40% to ~80%. This slowing down of evolutionary rates can also arise from the heterogeneity of fitness effects of mutations^8^.

## Results

### Proteome-wide espistasis in *E. coli*

**Figure 1:**
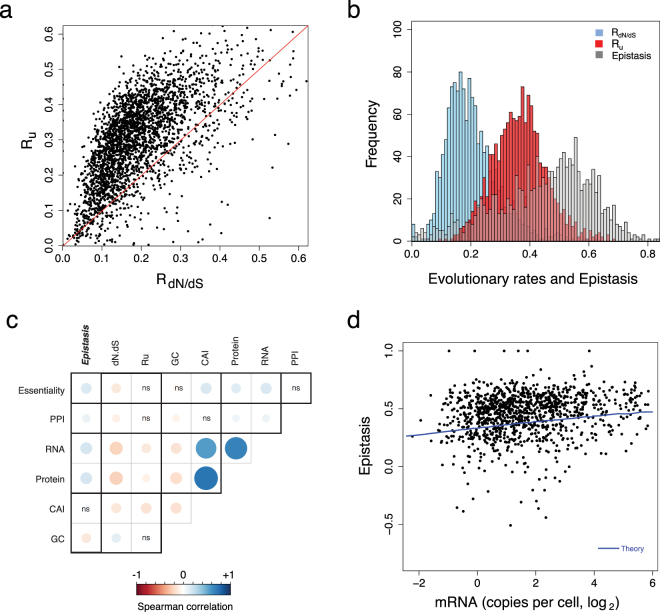
Proteome-wide estimate of epistasis in *E. coli*. (**a**) Background-dependent evolutionary rate *R*_*dN/dS*_ is significantly slower than the background-independent rate of mutational usage *R*_*u*_ (Wilcoxon signed-rank test, p-value < 10^−16^). (**b**) The average epistasis is ~41 ± 16% among 2,382 genes in *E. coli*. (**c**) Epistasis is positively correlated with genome-wide factors: mRNA and protein expression levels, essentiality of proteins, number of protein-protein interactions and codon adaption index (CAI) (see Table S4 for the correlation coefficients and p-values). Boxes labeled “ns” are not significant (p-value > 0.05). (**d**) Highly expressed genes experience strong epistasis (Spearman r = +0.17, p-value < 10^−9^), which can be explained by a model of sequence evolution based on selection against protein misfolding and aggregation (blue line; see also Figs S3, S4 and S10).

To determine if epistasis is indeed experienced by all genes in a proteome we estimated epistasis in the evolution of 2,382 genes in *E. coli* using thousands of orthologs within the class *gammaproteobacteria* (see Methods and Supporting Information). We calculated *R*_u_ and *R*_dN/dS_ from the multiple sequence alignment of each gene (Methods). The rates *R*_u_ and *R*_d*N*/d*S*_ and the epistasis for each gene are shown in Fig. 1a and their distributions in Fig. 1b. Since epistasis is expected to slow down the rate of evolution, the lineage-independent rate *R*_*u*_ is greater than the lineage- and background-dependent *R*_d*N*/d*S*_ (note the deviation from R_u_ = R_dN/dS_ line in Fig. 1a; Wilcoxon signed-rank test, p-value <10^−16^). The average *R*_u_ and *R*_d*N*/d*S*_ are 0.36 ± 0.09 and 0.20 ± 0.08, respectively, which lead to a proteome-wide epistasis of ~41% (Fig. 1b; full data is listed in Table S1). This estimate implies that epistasis and background specificity of mutational effects in proteins slows down the evolutionary rates of proteins in *E. coli*, on average, by ~41%. The magnitude of epistasis is broadly distributed with some genes experiencing epistasis of up to ~80% (Fig. 1b). These estimates over several thousand genes is slightly lower than the value calculated by Breen *et al*.^6^ for 16 mammalian proteins.

Next, we checked for the robustness of our results to factors that may influence the calculation of substitution rates and epistasis. First, the evolutionary rates, in particular *R*_*u*_ that count the number of unique amino acids, are sensitive to the number of orthologs in an MSA. Too few orthologs may lead to undersampling of *R*_*u*_ and to negative values for epistasis. However, as shown by the plot of epistasis versus the number of orthologs (Fig. S1), this artifact is present only in genes with MSA alignments less than 200 orthologs. Second, a distinction should be made between fixed and non-fixed amino acids. In principle, fixed amino acids are substitutions that are kept in long-term evolution while non-fixed amino acids are segregated in the population at short time scales and are eventually lost. Since Equation 1 estimates epistasis in long-term protein evolution, non-fixed amino acid states or polymorphisms can inflate the mutational usage *R*_*u*_ and epistasis. To account for the bias due to non-fixed polymorphic states in our amino-acid usage calculation, we used a correction based on the probability of occurrence of non-fixed amino acids at given site in the alignment (Fig. S2 and Supporting Information). The average correction to *R*_*u*_ due to non-fixed polymorphism is only ~±2% (Fig. S2). Table S2 presents amino acid usage correction along with the probability of observing a non-fixed state as fixed for all genes. Lastly, the calculation of *R*_d*N*/d*S*_ could be sensitive to the counting method. We control for this effect by using several counting methods (five heuristic and two maximum-likelihood codon-based approaches) for *dN*, *dS*, and *dN*/*dS* (Figs S5, S6, S7 and Table S3) and chose the most unbiased (Methods). Altogether, our estimates of epistasis are robust to the number of orthologs, presence of polymorphisms, and approaches for counting substitution rates.

### Relationship of epistasis with genomic properties

The rate of protein evolution is influenced by several factors ranging from molecular, to cellular, and to population level^9^. We determined if epistasis is also influenced by these factors. Specifically, we calculated the correlation between epistasis and publicly available data on mRNA expression level, protein abundance, gene essentiality, protein-protein interaction (PPI), and codon adaptation index (CAI) (Fig. 1c, Table S4 see Methods). We found that epistasis shows a weak yet significant positive correlation with number of orthologs, PPI, mRNA and protein expression levels, as well as CAI. As shown previously^10^, *R*_d*N*/dS_ negatively correlates with expression level (*r* = −0.24, p-value < 10^−16^) implying that highly expressed genes are under strong purifying selection. Since *R*_*u*_ also reflects selection, it similarly shows negative correlation with expression level (*r* = −0.18, p-value < 10^−10^). However, the weaker anti-correlation between *R*_*u*_ and expression level compared to that of *R*_d*N*/dS_ leads to a positive correlation between epistasis and expression level (*r* =  + 0.17, p-value < 10^−9^) (Fig. 1d). This finding implies that background specificity significantly slows down the rate of evolution among highly expressed genes.

To further check the biological significance of proteins evolving under high epistasis, we performed Gene Ontology (GO) enrichment analyses^11^ on the lowest and highest quantiles of epistasis (see Table S5). The lowest quantile corresponds to genes with epistasis less than 18% and lower, and the top quantile with epistasis greater than 53%. Interestingly, essential processes such as amino acid and nucleobase synthesis, ATP and RNA binding and proteostasis were significantly enriched among the genes evolving with high epistasis (Table S6). For example, the genes *glyA* and *prs* encoding the Serine hydroxymethyltransferase and Ribose-phosphate pyrophosohokinase, evolve with ~65% epistasis with ~1000 orthologues and are essential enzymes in the synthesis of amino acids and nucleobases. Other examples are the chaperone protein DnaK with 63% (944 orthologues) and elongation factor 4 (EF4) with 67% epistasis (953 orthologues). In contrast to highly epistatic genes, those evolving with low epistasis are mainly transmembrane proteins (Table S7). It is well-established that membrane proteins are dramatically less conserved that water soluble proteins and have higher evolutionary rates due to adaptation to the changing environment^12^ which could then influence the estimated epistasis using Eq. 1. Altogether, our proteome-wide estimates demonstrate that highly expressed genes not only experience stronger purifying selection, but also greater epistasis in their long-term evolution. This result highlights the coupling of selection and epistasis in proteome evolution^13^.

### Model of sequence evolution based on protein folding explains proteome-wide correlation between epistasis and expression level

The negative correlation between evolutionary rate *R*_dN/dS_ and expression level is well-established^10,14,15^. This observation has been explained by a model of sequence evolution based on selection against protein misfolding due to mistranslation^10^ or genetic mutations^14,16^. The biological rationale is that misfolded proteins can form aggregates  that are toxic to the cell^17,18^. To determine if the same hypothesis can quantitatively explain the trend between epistasis and mRNA expression level, we combine the population genetic formalism for evolutionary rate with protein folding thermodynamics^14,19–21^. Assuming that cellular fitness *F* is *inversely* proportional to the total number of misfolded proteins in the cell, it may be formally written as^10^:

2 $$F=exp[-c(\#\,of\,misfolded\,proteins)]=exp[-c(\sum _{k}^{{\rm{\Gamma }}}{A}_{k}\frac{1}{1+\exp (\beta {\rm{\Delta }}{G}_{k})})]$$

Equation 2 expresses the probability that the protein product of gene *k* is unfolded as a function of its stability Δ*G*_*k*_. The energy factor *β* = 1/*k*_*b*_ *T* where *k*_*b*_ *T* ~ 0.59 kcal/mol at room temperature. This probability multiplied by the cellular abundance of the gene *A*_*k*_ gives the number of misfolded copies (Fig. S10). The summation extends over all genes *Γ* in the proteome. The parameter *c* is the fitness cost of each misfolded protein (~10^−7^) (ref.^18^). As shown previously^10,14^ and in our specific dataset (Figs S3 and S4), this fitness function recapitulates the trend between d*N*/d*S* and expression level. But to arrive at epistasis, we also need a theoretical estimate for the mutational usage *R*_*u*_. In a recent work^19^, we showed that *R*_*u*_ is the rate of evolution of the most stable sequence in an MSA. By simulating sequence evolution (Supporting Information), we can arrive at a theoretical MSA evolved under the fitness function (Eq. 2) and then calculate *R*_*u*_ (Fig. S4).

Highly expressed (and more abundant) genes are under strong purifying selection; thus, *R*_*u*_ and *R*_*dN/dS*_ negatively correlate with mRNA level, both in theory (Fig. S4) and in *E. coli* (Fig. S3). More interestingly, the theoretical dependence of *R*_*u*_ vs. mRNA is weaker than *R*_*dN/dS*_ vs. mRNA leading to stronger epistasis among highly expressed genes (Fig. S4). Thus, selection against protein misfolding can explain the genomic observation that highly expressed and more abundant genes experience stronger epistasis (Fig. 1c,d). A geometric interpretation of epistasis is the curvature of the fitness landscape; indeed, for the genotype-phenotype relationship based on folding stability (Eq. 2), the landscape exhibits greater curvature at higher expression levels (Fig. S10).

## Discussion

Our study, for the first time, provides proteome-wide estimate of epistasis in *E. coli*. On average, a protein in *E. coli* evolves with ~41% epistasis. One interpretation of this result is that the rate of protein evolution is reduced by 41% due to background dependence of mutational effects. Moreover, we found that highly expressed proteins evolve with stronger epistasis, which can be explained by selection against protein misfolding. Our results highlight the coupling between selection and epistasis, which has been demonstrated in specific proteins^4,22^, but not in the long-term evolution of a proteome.

We also tested the enrichment of functional groups among genes under high or low epistasis. We found that genes evolving with high epistasis are involved in essential processes such as the maintenance of proteostasis and rRNA and ATP binding. This finding is in line with previous observations that genes with high intergenic pleiotropy in yeast are often involved in more cellular processes than low pleiotropic genes^23^. Here we systematically showed that intragenic epistasis has the same pattern in *E. coli*. We anticipate that future studies on the molecular evolution of proteins evolving with high epistasis could provide a mechanistic understanding of epistasis at the residue level. Furthermore, genes evolving with high epistasis are noteworthy as they tolerate maximum number of novel amino acids and thus are highly evolvable. The methodologies employed in this study can aid in selecting such genes at a genome-wide level. In addition, the coupling between epistasis, abundance and essentiality as described in this work can be used to update substitution matrices and phylogenetic trees of highly expressed proteins.

The extent of epistasis as reported in this work depends on amino acid usage and evolutionary rate of proteins, and both quantities were shown to vary in different habitats and can be influenced by environmental conditions^24,25^. To investigate the role of habitat and lifestyle on epistasis, we estimated epistasis in the evolution of *E. coli* orthologous proteins within the two classes of *alpha-* and *betaproteobacteria*. Bacterial species within the classes *alpha* and *betaproteobacteria* generally live in low and high nutrient environments, respectively^26,27^. We retrieved 2098 orthologs for *E. coli* proteins within the class *alphaproteobacteria* and 2174 within the classes *betaproteobacteria*. We then compared epistasis across the three classes, while controlling for the unequal number of orthologues (see Methods).

**Figure 2:**
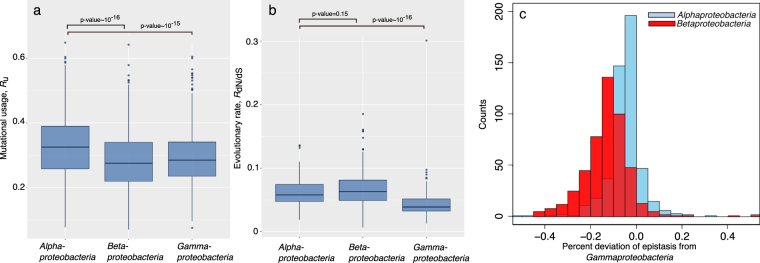
Epistasis is influenced by bacterial lifestyles. (**a**) *R*_u_ and (**b**) *R*_dN/dS_ in the evolution of *E. coli* orthologous proteins within the three classes of *alpha-*, *beta-* and *gammaproteobacteri*. In (**c**) the percent deviation of epistasis in the evolution of *alpha-* and *betaproteobacteri*a from *gammaproteobacteria* is calculated. All the p-values are calculated using Wilcoxon rank sum test.

As shown in Fig. 2a, the average *R*_u_ is slightly higher for orthologs in *alphaproteobacteria* (<*R*_*u*_> = 0.33 ± 0.09) than *betaproteobacteria* (<*R*_*u*_> = 0.28 ± 0.09) and *gammaproteobacteria* (<R_u_> = 0.29 ± 0.09) with p-values of ~10^−16^ (Wilcoxon signed-rank test). Evolutionary rate, *R*_dN/dS_, when corrected for the level of divergence (d*S*), is not significantly different (p-value = 0.15) between *alpha* and *betaproteobacteria* with <R_d*N*/d*S*_> = 0.062 ± 0.023 and 0.068 ± 0.032, respectively (Fig. 2b). This makes the average epistasis ~0.76, 0.67 and 0.80 for *alpha*, *beta* and *gammaproteobacteria*. Note that epistasis is overestimated (i.e., compared to previously reported 41%) due to the smaller number of orthologues in this comparison. Therefore, proteins in *alpha* and *betaproteobacteria* evolve with 5% and 16% lower epistasis compared to their orthologs in *gammaproteobacteria* mainly because of differences in mutational usage (Fig. 2c). As elegantly showed by Akashi and Gojobori, mutational usage is significantly different in bacteria with different metabolic profiles^28^. We anticipate that biosynthetic cost minimization, among other factors, may underlie the differences in the extend of mutational usage and hence epistasis in the evolution of proteins within these classes of *proteobacteria* phylum.

This work focused on the *E. coli* proteome; however, it will be interesting to generalize these observations in other well-studied model organisms such as yeast, worm, fly, mouse, and human, where selection (detected by d*N*/d*S*) has been shown to strongly correlate with expression level^10^. Demonstrating these results across all kingdoms of life could generalize the finding that selection due to folding stability is a universal mechanism for some of the epistasis experienced in the long-term evolution of a proteome.

## Methods

### Sequences and alignment

List of genes for *Escherichia coli* K-12 MG1655 was taken from NCBI. From this list (4140 genes), KEGG ids (total of 3059 ids) were used to retrieve functional orthologs within *Gammaproteobacteria* class. Ortholog sequences were available for 2814 of the 3059 genes. To optimize alignment, sequences 15% longer or shorter from the reference *E. coli* gene were removed from the set. DNA sequences were converted to protein sequences prior to alignment and calculation of amino-acid usage. For the protein alignments, we used default parameters except for the allowed positions with gaps that were set to half, to allow gaps at positions where less than 50% of sequences had gaps.

### Bioinformatics

The amino-acid usage measure can be used to obtain an estimation of d*N*/d*S* ratio under the assumption of non-epistatic evolution. The amino-acid usage <*u*> is defined as the number of different amino-acids observed at one site, averaged over all sites in an alignment. We can then estimate non-epistatic d*N*/d*S* from <*u*> using (*u* − 1)/19 where (*u* − 1) is the number of amino-acid states into which the current amino acid can be substituted, divided by 19 amino-acid possibilities. The choice of a proper and unbiased method to estimate d*N*/d*S* is crucial in the current work. We thus systematically checked performance of five different heuristic counting approaches and two maximum-likelihood (ML) codon models for 3124 genes in *E. coli* and concluded that the simplest model of Nei and Gojobori^29^ gives the most unbiased d*S* and d*N*/d*S* estimates which reasonably fit values from accurate yet computationally expensive ML methods. For the complete analysis check Supplementary methods and Table S4. All the GO-enrichment analyses were done using DAVID bioinformatics resources^30^.

To compare epistasis among the three classes of *alpha-*, *beta-*, and *gammaproteobacteria*, we focused on proteins with more than 300 orthologs within each class. This number was chosen to insure we have more than 500 genes and thus the proteome-wide estimate of epistasis is within 95% confidence level and a margin of error of less than five percent^31^. We then estimated R_u_, d*N*, d*S* and *R*_dN/dS_ for the exact number of 300 orthologs for each gene. When more orthologs within each class were available, we randomly selected 300 sequences and calculated the average of all quantities for ten repeats. As the number of orthologs are significantly smaller for the classes *alpha-* and *betaproteobacteria*, *R*_u_ is underestimated for proteins in these classes. As a result, epistasis would be overestimated for orthologous proteins in *alpha-* and *betaproteobacteria*. To resolve this issue, we applied the same procedure for *E. coli* proteins within the class *gammaproteobacteria* and calculated evolutionary rates and epistasis.

### Theoretical model

To calculate the extent of epistasis, we used an expression for substitution rates that takes the stability effects of mutations into account (Equation 1). Fitness is proportional to the number of misfolded copies in the cell which in turn is a product of total abundance and the probability of being in the folded state. This decomposition enables us to utilize the known distribution of mutational effects on protein stability to determine the distribution of fitness effects and calculate evolutionary rates accordingly (see Supplementary information for full analysis). The fitness landscape (Equation 2) contains protein abundance, thus we  converted mRNA abundance to protein abundance using their well-established correlation (Supplementary information).  The calculation of the rate of mutational usage *R*_*u*_ and pairwise rate of evolution *R*_dN/dS_ based on the fitness landscape of Equation 2 is described in the Supplementary information.

## Electronic supplementary material

Supporting Information

Table S1

Table S2

Table S3

Table S4

Table S5

Table S6

Table S7
