# Supplementary material for: Highly expressed genes evolve under strong epistasis from a proteome-wide scan in E. coli
Source: Sci Rep. 2017 Nov 20;7:15844. doi: 10.1038/s41598-017-16030-z (PMC5696520; doi:10.1038/s41598-017-16030-z)
Supplement: Supplementary file 1 — Supporting Information [file 41598_2017_16030_MOESM1_ESM.pdf]

**Supplementary information**

# Highly expressed genes evolve under strong epistasis from a proteome-wide scan in *E. coli*

Pouria Dasmeh[1,2, #], Eric Girard[1,2, #], Adrian W.R. Serohijos[1,2, *]

[1]Departement de Biochimie, [2]Centre Robert Cedergren en Bioinformatique et Génomique, Université de Montréal, 2900 Edouard-Montpetit, Montréal, Quebec H3T 1J4, Canada

[*]*Correspondence: adrian.serohijos@umontreal.ca*
[#]Equal contribution.

## Supplementary methods:

**Sequences:**

The list of genes for *Escherichia coli* K-12 MG1655 was taken from NCBI. We mapped these genes to their ids from the KEGG database[1]. We then used the KEGG ids to get ortholog sequences. Orthologs were available for 2814 of the 3059 genes within the *Gammaproteobacteria* class.

**Alignment:**

To optimize alignment, orthologs with sequences 15% longer or shorter than the reference *E. coli* gene were removed from the data set. DNA sequences were converted to amino acid sequences prior to alignment for the calculation of amino-acid usage *u*. The multiple alignments were constructed using MUSCLE[2], which uses distance measures and guide tree estimation followed by a progressive alignment. Gblocks [4] was used to remove gaps in the alignment resulting from poorly aligned positions and divergent regions. For the protein alignments, we used default parameters.

**Estimation of amino-acid usage:**

The amino-acid usage *u*, similar to *dN*/*dS*, reflects the stringency of selection. However, unlike *dN*/*dS*, which is measured over the length of a gene, *u* is measured per site. Thus, *dN*/*dS* reflects background dependence while *u* does not[3]. The amino-acid usage *u* is defined as the number of different amino acids observed at one site, averaged over all sites in an alignment. We estimated

non-epistatic rate of substitution as using ($u$-1)/19 where ($u$-1) is the number of amino-acid states to which the current wildtype can transition, normalized by all possible substitutions (20-1=19).

**Calculation of d*N*/d*S*:**

We obtained codon alignments with PAL2NAL[4]; it uses a multiple protein sequence alignment and their corresponding DNA sequences as input. We then used Gblocks as described above but we remove all positions with gaps.

The estimation of d*N*/d*S* is sensitive to the choice of counting methods and to potential saturation in synonymous substitution rate or d*S*>1. Given the essential role of averaged pairwise d*N*/d*S* in the estimation of epistasis (**Eq. 1** in the main text), a careful analysis of d*N*/d*S* is required. We used seven different methods to estimate d*N*/d*S* for the 3124 genes:

1. NG: Nei, M. and Gojobori, T. (1986)[5]
2. LWL: Li, W.H., et al. (1985)[6]
3. LPB: Li, W.H. (1993)[7] and Pamilo, P. and Bianchi, N.O. (1993)[8]
4. MLWL (Modified LWL): Tzeng, Y.H., et al. (2004)[9]
5. MLPB (Modified LPB): Tzeng, Y.H., et al. (2004)[9]
6. YN: Yang, Z. and Nielsen, R. (2000)[10]
7. MYN (Modified YN): Zhang, Z., et al. (2006)[11]

The first five (NG, LWL, LPB, MLWL, and MLPB) are heuristic counting methods and the last two (YN and MYN) are the maximum-likelihood codon-based methods. The main difference between YN and MYN model is the choice of codon frequency that in MYN is estimated from the product of the average observed nucleotide frequencies in the three codon positions (F3X4). We used KaKs_Calculator[12] to estimate d*N*/d*S* for all models except MYN which was estimated by CODEML program within the PAML suite[13]. Estimation of d*N*/d*S* using ML-based codon models is computationally infeasible for all genes and their orthologs in our study (a total of 573,545,811 pairwise comparisons). We thus estimated d*N*/d*S* using all heuristic counting models between *E.coli* and *Salmonella enterica* ortholog genes (3124 comparisons) and compared these methods with the MYN model to find the most unbiased yet computationally tracktable method. **Figures S5, S6** and **S7** show d*S*, d*N* and d*N*/d*S*, respectively, for all genes

calculated using the five heuristic methods (NG, LWL, LPB, MLWL, MLPB) and YN against the more accurate MYN model. From **Fig. S6**, d*N* values are almost identical in all methods suggesting that the *dN* values are robust.

In the case of *dS*, all methods show substantial variation at values >1. More importantly, d*S* is underestimated in all Li-based method (LWL, LPB, MLWL and MLPB) which commonly correct for transition/transversion bias. These results are in full agreement with the basic theory of the role of transition/transversion bias in estimating d*N*/d*S*. Since transitions are more likely silent mutations than transversions, ignoring the transition/transversion rate differences results in overestimation of dS[6]. **Fig. S10** shows this trend in our dataset when *dS* estimated from *NG* method (assuming no transition/transversion bias) is compared with *dS* estimated using LWL. Another factor to be considered is the unequal codon frequencies in the MYN model compared to other methods. As thoroughly discussed by Yang and Nielsen[10], unequal codon frequencies would have the opposite effect to the transition/transversion bias. Therefore, all Li-based models that lack such effect would underestimate *dS*. Altogether, this shows that the NG method which lacks both effects (transition/transversion and unequal codon biases) would produce the most unbiased estimate of epistasis compared to Li-based methods. All estimated rates are compiled in **Table S3**.

**<u>Amino-acid usage correction for non-fixed states:</u>**

In principle, polymorphisms, or non-fixed states, in a species can potentially inflate the counting of amino acid usage. To estimate the impact of non-fixed states in our amino-acid usage calculation, we used a correction based on the probability of occurrence of non-fixed amino acids $p$ [3]. To calculate $p$, we first measured the average amino acid diversity $\prod_a$ for each gene. The amino acid diversity is a measure of the fraction of amino acid mismatches between two sequences of the same species in a pairwise alignment. We calculated the average $\prod_a$ values for each species with at least two sequences, and then averaged $\Pi_a$ values across all species. Since amino-acid diversity is roughly the expected density of non-fixed states, the probability of observing a non-fixed state $p$ is $\prod_a/6$. The factor of 6 is the number of possible non-synonymous

mutations away from an average codon.

If non-fixed states have a probability of $p$, then fixed states have a probability of $q = 1 - p$ and the distribution of those states in a multiple alignment follows a binomial distribution. To approximate the probability that a non-fixed state is observed $k$ times in an alignment of $N$ sequences, we use the Poisson formula $m=(p\mathrm{N})^{k}e^{-p\mathrm{N}}/k!$. The probability to have a fixed, rather than non-fixed, is then $r = 1 - m$. Finally, we take the sum of the probabilities of fixed-states for each amino-acid state observed at one site or $\sum_{i=1}^{u} r_i$ , where $i$ is the amino-acid state and $u$ is the amino-acid usage. We further average this sum across all sites of the gene to get the corrected $u$. The average correction to $u$ is $\Delta u \sim 4\%$ (values for each gene are listed in **Table S2**). Consequently, the correction to $R_u$ due to polymorphism is $\Delta R_u = \left(\frac{1}{L}\right)\left(\sum_i \frac{\Delta u}{19}\right) \approx 2\%$ (**Fig. S2**).

**Selection against misfolding toxicity:**

Under selection against misfolding toxicity, as originally proposed by Drummond and Wilke[14], the fitness of a cell is negatively proportional to the number of misfolded proteins $[U]$:

$$Fitness \sim \exp(-c[U]) \quad \textbf{(Equation S1)}$$

where $c$ is the fitness cost imposed by each misfolded protein and is shown to be $\sim 10^{-7}$ in yeast. The number of misfolded proteins is the protein abundance $A$ multiplied by the probability of each single protein to be unfolded $P_{unfolded}$. Based on protein folding thermodynamics and assuming equilibrium between folded and unfolded state, the probability of being unfolded is $P_{unfolded} = 1/\left(1+e^{\beta \Delta G}\right)$, where $\Delta G$ is the stability of the protein quantified by its folding free energy. The energy factor $\beta = 1/k_b T$ where $k_b T \sim 0.59$ kcal/mol at room temperature. Fitness can then be written as

$$Fitness = \exp\left[-cA\left(\frac{1}{1+\exp(\beta \Delta G)}\right)\right] \quad \textbf{(Equation S2)}$$

Selection coefficient of an arising mutation can be expressed as:

$$s = \ln(F_{mut}) - \ln(F_{WT}) = -cA\left(\frac{1}{1+e^{-\beta(\Delta G_{mut}=\Delta G_{WT}+\Delta\Delta G)}} - \frac{1}{1+e^{-\beta(\Delta G_{WT})}}\right) \quad \textbf{(Equation S3)}$$

Here the effect of mutations on protein folding stability is assumed to be additive, i.e., $\Delta G_{mut} = \Delta G_{WT} + \Delta\Delta G$ where $\Delta\Delta G$ is the change in protein folding stability upon mutation. Despite this assumption on the additive effect of mutations on stability (demonstrated to be true for some proteins[15]), there is epistasis in the fitness landscape model because the mapping between stability and fitness is non-linear (**Fig. S10**).

The substitution rate of an arising mutation is then calculated using the selection coefficient from **Eq. S3**:

$$\omega = N\,P_{fix} = N\frac{1-e^{-2s}}{1-e^{-2Ns}} \quad \textbf{(Equation S4)}$$

where $N$ is the effective population size. **Eq. S4** is the $dN/dS$ for a random mutation that changes the wildtype folding stability, $\Delta G_{WT}$, by an amount $\Delta\Delta G$. $R_{\mathrm{dN/dS}}$ as defined in the main text is $dN/dS$ averaged over all arising mutations and multiple backgrounds (multiple orthologs in an MSA), thus

$$R_{dN/dS} = \iint N \frac{1-e^{2cA\left(\frac{1}{1+e^{-\beta(\Delta G_{WT}+\Delta\Delta G)}} - \frac{1}{1+e^{-\beta(\Delta G_{WT})}}\right)}}{1-e^{2NcA\left(\frac{1}{1+e^{-\beta(\Delta G_{WT}+\Delta\Delta G)}} - \frac{1}{1+e^{-\beta(\Delta G_{WT})}}\right)}} P(\Delta G)P(\Delta\Delta G)d(\Delta G)d(\Delta\Delta G) \quad \textbf{(Equation S5)}$$

The distribution $P(\Delta\Delta G)$ is the probability distribution of mutational effects on folding stability known from large-scale mutational studies[16-19], and has been parameterized for proteins of different folds. The distribution of background folding stability $P(\Delta G)$ is a consequence of mutation-selection balance on the protein folding fitness landscape[20-24]. This distribution has also been documented experimentally from >4000 proteins[25].

To calculate the rate obtained from mutational usage $R_u$, we assume that each protein sequence in an MSA corresponds to a $\Delta G$ value in $P(\Delta G)$[26]. Moreover, in the regime where proteins are very stable (**Fig. S10**), the fitness landscape is flat with respect to folding stability, which implies that

the current amino acid can be substituted by all other amino acids, that is, $u$=19. In the regime of marginal stability, the presence of selection implies that amino acids are not allowed, that is, $u$<19. Thus, $R_u$ is the rate of evolution of the most stable sequence in an MSA[26]. In the case of continuous distribution of $P(\Delta G)$, $R_u$ is the rate at the probability cutoff, e.g., $P(\Delta G) = 0.01$ or

$$R_u = \int \omega(\Delta G = \min(\Delta G|_{P(\Delta G)=0.01}), \Delta\Delta G) P(\Delta\Delta G) \quad \textbf{(Equation S6)}$$

The meaning of 0.01 is that the probability of sampling this specific $\Delta G$ is one in 100 trials. We have shown that $R_u$ calculated using this approach shows an excellent correlation with $R_u$ calculated directly from multiple sequence alignment[26].

**<u>Theoretical prediction of relation between epistasis and expression level</u>**

We calculate epistasis at different mRNA stabilities using the following steps:

1. An mRNA expression level is chosen from the set of measured expression levels for different *E. coli* genes (**Table S1**).
2. The corresponding protein expression level (**Table S1**) is used in **Eqns. S1-S7**
3. We assumed $c$=$10^{-7}$ (ref. [27]).
4. Distribution of mutational effects on protein folding stability, $P(\Delta\Delta G)$, was assumed to be
   $$P(\Delta\Delta G) = p_1 \mathbb{N}(\mu_1, \sigma_1^2) + (1 - p_1)\mathbb{N}(\mu_2, \sigma_2^2) \quad \textbf{(Equation S7)}$$
   where $p_1$ is the weights of first distribution, $\mu_1$, $\mu_2$, $\sigma_1$ and $\sigma_2$ are the average values and standard deviations of each Gaussian distribution found to be 0.53 ± 0.12, 0.56 ± 0.12, 1.96 ± 0.53, 0.90 ± 0.16 and, 1.93 ± 0.29 respectively[16].
5. The distribution of mutational effects on protein folding stability, **Eq. S7**, is not assumed constant at all stabilities. We used the following empirical formula[23,25,28]:
   $$\Delta\Delta G_{mean} = -0.13(\Delta G) + 0.23 \quad (kcal/mol) \quad \textbf{(Equation S8)}$$
   **Eq. S7** implies that at higher stabilities fewer stabilizing mutants would be available to a protein, as confirmed from large-scale experimental observations[25].
6. Distribution of protein stabilities, $P(\Delta G)$, was taken from that of experimentally-calibrated log-normal distribution for *E. coli* [29] with following parameters:

$$\langle \Delta G \rangle = -7.10 \quad (kcal/mol)$$
$$\sigma(\Delta G) = 2.53 \quad (kcal/mol)$$

7. $R_{\mathrm{dN/dS}}$ is then calculated from **Eq. S6** taking the knowledge of fitness function and probability distribution, $P(\Delta G)$ and $P(\Delta\Delta G)$.
8. $R_{\mathrm{u}}$ is calculated from **Eq. S7**.
9. Epistasis is calculated from **Eq. 1** as $\varepsilon = 1-(R_{dN/dS})/(R_u)$. The steps are repeated for a different mRNA-expression level.

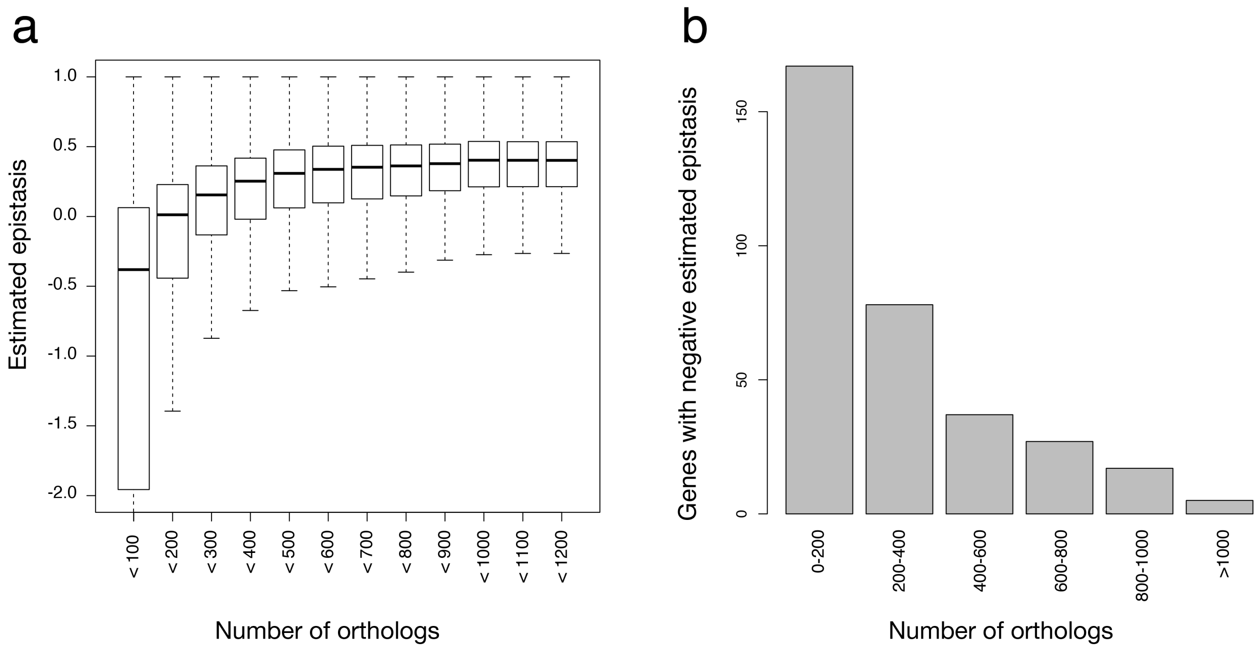


**Figure S1**. Genes with negative epistasis is due to few numbers of orthologs. **(a)** Estimated epistasis versus number of orthologs in an MSA. **(b)** Number of genes with negative epistasis as a function of number of orthologs.

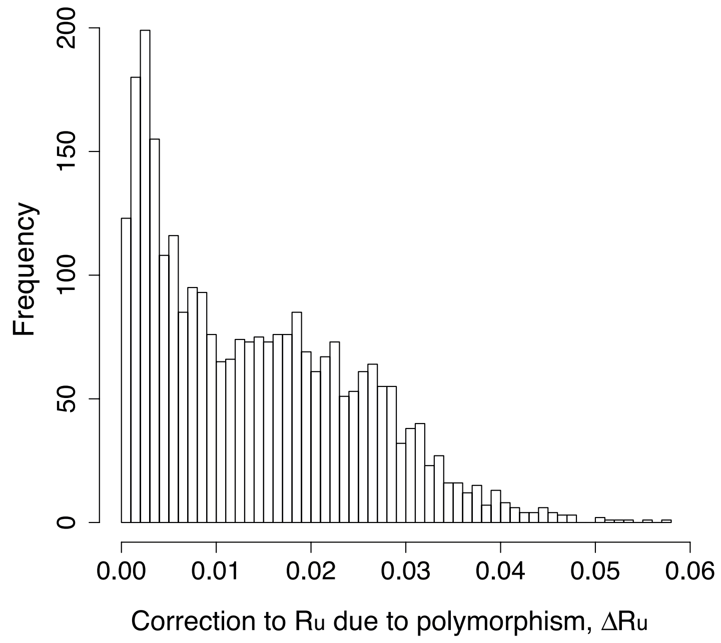


**Figure S2**. A correction to mutational usage, *u*, is accounted for by estimating for each gene the probability of counting a non-fixed polymorphism as fixed state (**Table S2**). The average correction to *u* is $\Delta u \sim 4$ %. Consequently, the correction to $R_u$ due to polymorphism is $\Delta R_u = \left(1/L\right)\left(\sum_i \Delta u/19\right) \approx 2\%$.

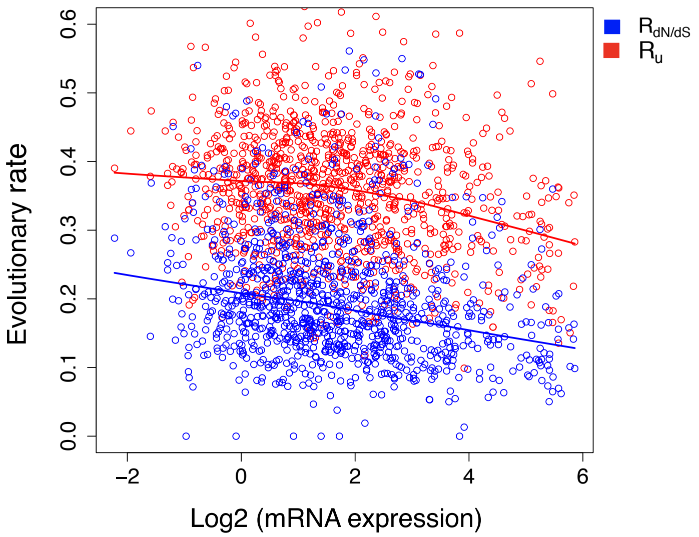


**Figure S3**. $R_u$ and $R_{dN/dS}$ as a function of mRNA expression level in *E. coli*. Lines show the Lowess fit to data.

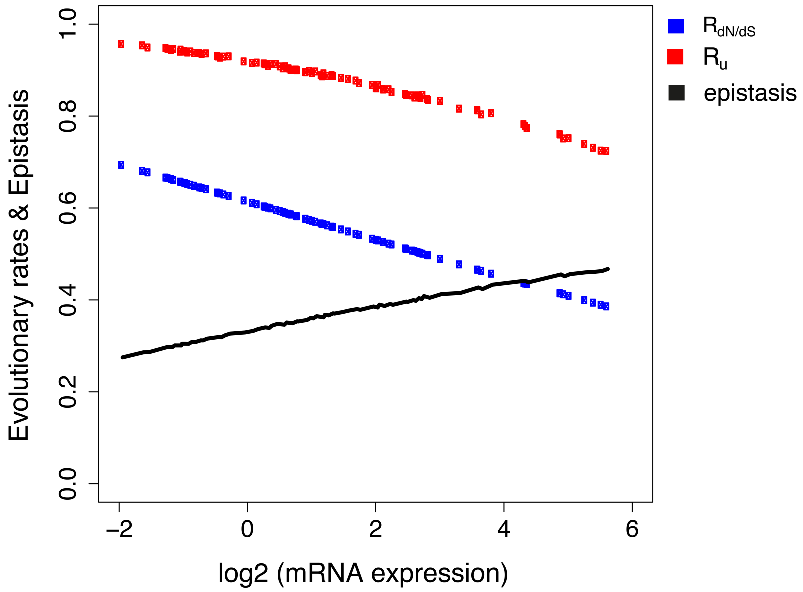


**Figure S4**. Theoretical $R_u$, $R_{dN/dS}$ and epistasis as a function of mRNA expression level based on a model of sequence evolution where fitness is inversely proportional to the number of misfolded copies of proteins in the cell (**Eq. 2 & Fig. S10**). Effective population size in the theoretical calculations is $10^6$. The theoretically estimated curve for epistasis is also shown in **Fig. 1d**.

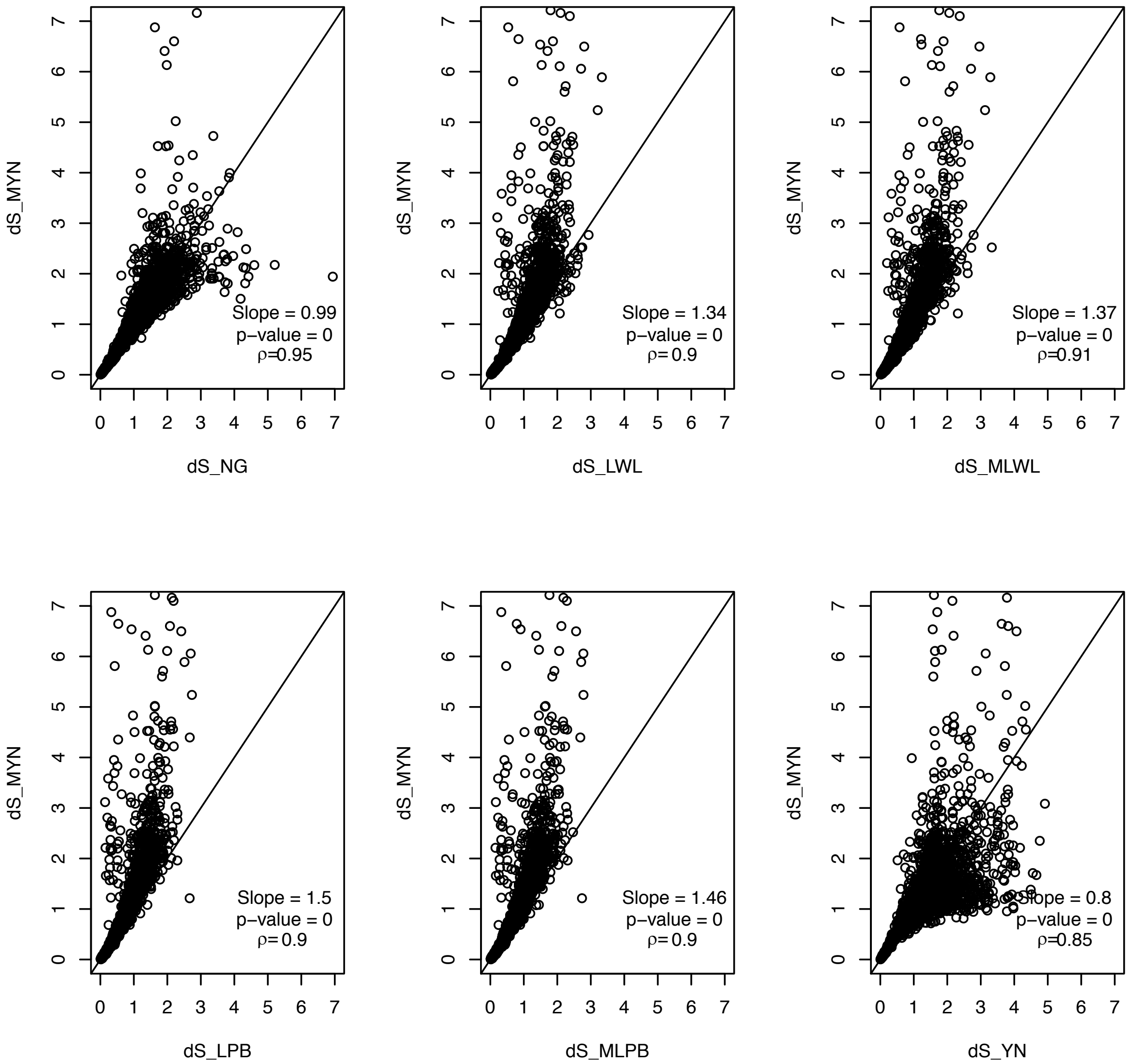


**Figure S5**. Comparison of normalized rate of synonymous substitution rate, d*S*, estimated from different heuristic (NG= Nei-Gojobori, LWL= Li, W.H., et al., MLWL=modified LWL, LPB= Li, W.H. (1993) and Pamilo, P. and Bianchi, N.O. (1993), MLPB=modified LPB) with maximum-likelihood based codon models (MYN). The model NG does not assume transition/transversion bias and unequal codon frequencies. All Li-based methods (LWL, MLWL, MPB and MLPB) accommodate transition/transversion but assume equal codon frequencies. Models YN and MYN both assume transition/transversion and unequal codon frequencies.

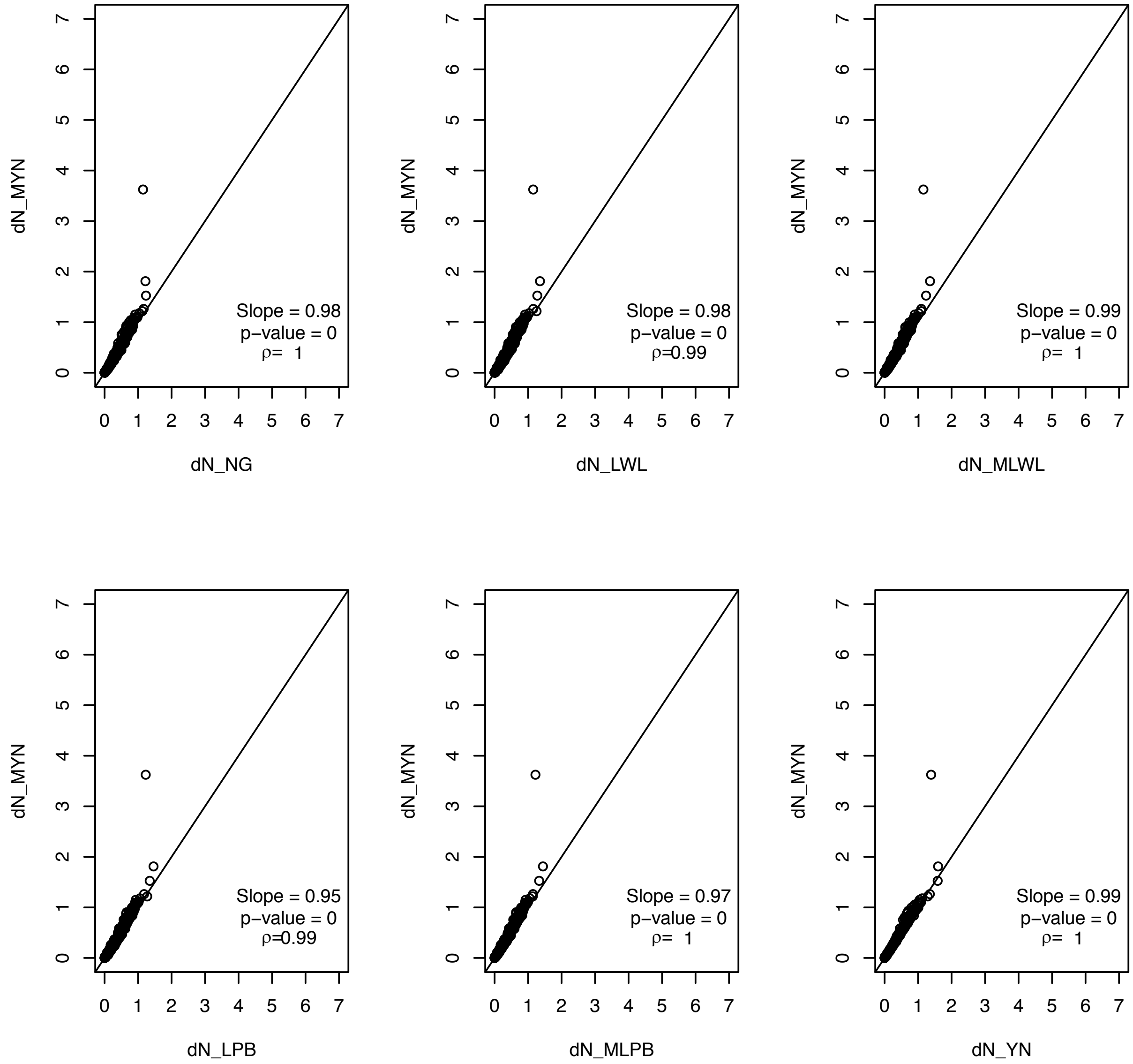


**Figure S6**. Comparison of normalized rate of nonsynonymous substitution rate, d*N*, estimated from different heuristic (NG= Nei-Gojobori, LWL= Li, W.H., et al., MLWL=modified LWL, LPB= Li, W.H. (1993) and Pamilo, P. and Bianchi, N.O. (1993), MLPB=modified LPB) with maximum-likelihood based codon models (MYN). The model NG does not assume transition/transversion bias and unequal codon frequencies. All Li-based methods (LWL, MLWL, MPB and MLPB) accommodate transition/transversion but assume equal codon frequencies. Models YN and MYN both assume transition/transversion and unequal codon frequencies. Nonsynonymous rates are less diverged/saturated thus different methods are in stronger agreement with respect to d*N*.

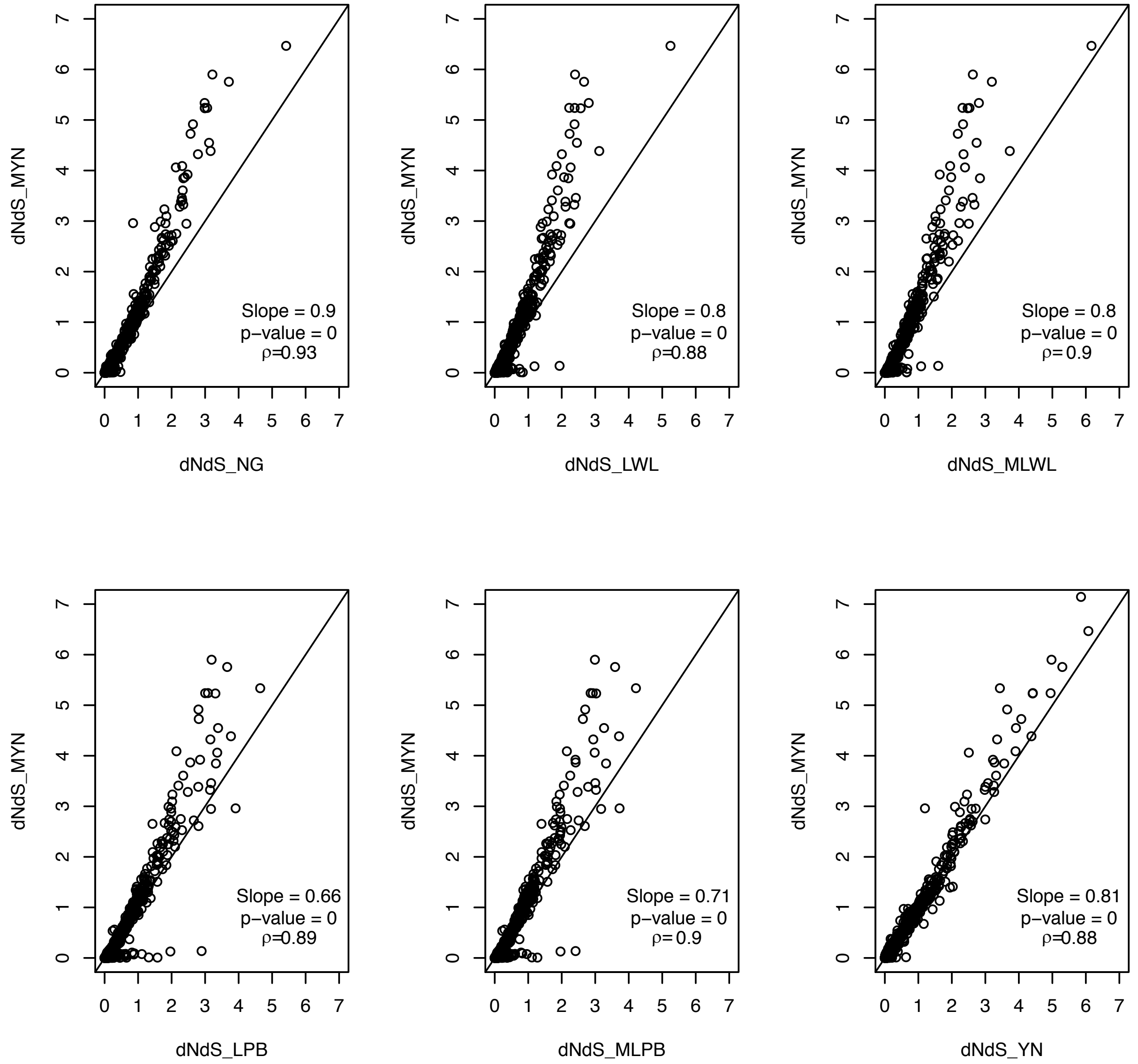


**Figure S7.** Comparison of the ratio of normalized rate of nosynonymous and synonymous substitution rates, d*N*/d*S*, estimated from different heuristic (NG, LWL, MLWL, LPB, MLPB) with maximum-likelihood based codon models (MYN). The model NG does not assume transition/transversion bias and unequal codon frequencies. All Li-based methods (LWL, MLWL, MPB and MLPB) accommodate transition/transversion but assume equal codon frequencies. Models YN and MYN both assume transition/transversion and unequal codon frequencies.

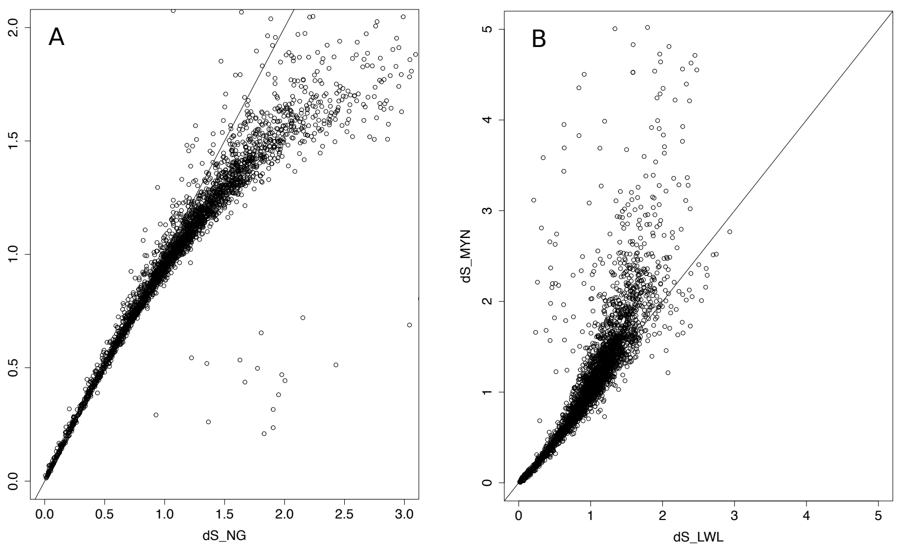


**Figure S8**. Comparison of d*S* A) between NG and LWL models and B) between LWL and MYN models.

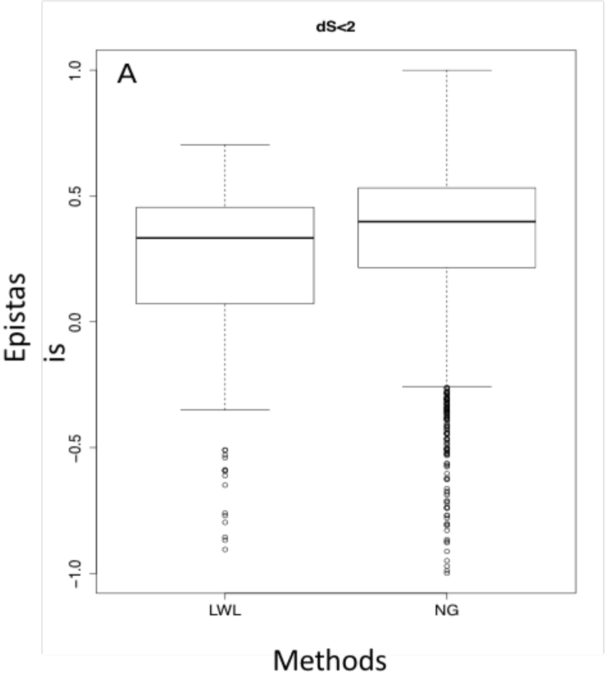


**Figure S9**. Controlling for d*S* saturation gives closer overall estimate of epistasis using NG and LWL models.

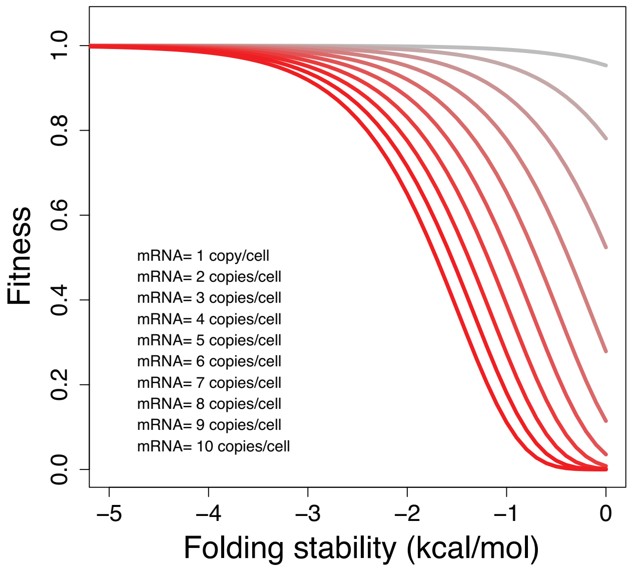


**Figure S10**. Fitness as a function of protein folding stability (**Eq. 1 and S2**) plotted for different mRNA expression levels. Highly expressed (abundant) proteins are under stronger selection (i.e., greater fitness drop) than lowly expressed proteins. Highly abundant proteins sit in the regime of the fitness landscape that has greater curvature, hence they experience stronger epistasis.

**References**


1 Kanehisa, M. The KEGG database. *silico simulation of biological processes* **247**, 91-103 (2002).

2 Edgar, R. C. MUSCLE: multiple sequence alignment with high accuracy and high throughput. *Nucleic acids research* **32**, 1792-1797 (2004).

3 Breen, M. S., Kemena, C., Vlasov, P. K., Notredame, C. & Kondrashov, F. A. Epistasis as the primary factor in molecular evolution. *Nature* **490**, 535-538 (2012).

4 Suyama, M., Torrents, D. & Bork, P. PAL2NAL: robust conversion of protein sequence alignments into the corresponding codon alignments. *Nucleic acids research* **34**, W609-W612 (2006).

5 Nei, M. & Gojobori, T. Simple methods for estimating the numbers of synonymous and nonsynonymous nucleotide substitutions. *Molecular biology and evolution* **3**, 418-426 (1986).

6 Li, W.-H., Wu, C.-I. & Luo, C.-C. A new method for estimating synonymous and nonsynonymous rates of nucleotide substitution considering the relative likelihood of nucleotide and codon changes. *Molecular biology and evolution* **2**, 150-174 (1985).

7 Li, W.-H. Unbiased estimation of the rates of synonymous and nonsynonymous substitution. *Journal of molecular evolution* **36**, 96-99 (1993).

8 Pamilo, P. & Bianchi, N. O. Evolution of the Zfx and Zfy genes: rates and interdependence between the genes. *Molecular Biology and Evolution* **10**, 271-281 (1993).

9 Tzeng, Y.-H., Pan, R. & Li, W.-H. Comparison of three methods for estimating rates of synonymous and nonsynonymous nucleotide substitutions. *Molecular biology and evolution* **21**, 2290-2298 (2004).

10 Yang, Z. & Nielsen, R. Estimating synonymous and nonsynonymous substitution rates under realistic evolutionary models. *Molecular biology and evolution* **17**, 32-43 (2000).

11 Zhang, Z., Li, J. & Yu, J. Computing Ka and Ks with a consideration of unequal transitional substitutions. *BMC evolutionary biology* **6**, 44 (2006).

12 Zhang, Z. *et al.* KaKs_Calculator: calculating Ka and Ks through model selection and model averaging. *Genomics, proteomics & bioinformatics* **4**, 259-263 (2006).

13 Yang, Z. PAML 4: phylogenetic analysis by maximum likelihood. *Molecular biology and evolution* **24**, 1586-1591 (2007).

14 Drummond, D. A. & Wilke, C. O. Mistranslation-induced protein misfolding as a dominant constraint on coding-sequence evolution. *Cell* **134**, 341-352 (2008).

15 Fersht, A. R., Matouschek, A. & Serrano, L. The folding of an enzyme. I. Theory of protein engineering analysis of stability and pathway of protein folding. *J Mol Biol* **224**, 771-782, doi:0022-2836(92)90561-W [pii] (1992).

16 Tokuriki, N., Stricher, F., Schymkowitz, J., Serrano, L. & Tawfik, D. S. The stability effects of protein mutations appear to be universally distributed. *Journal of molecular biology* **369**, 1318-1332 (2007).

17 Alber, T. Mutational effects on protein stability. *Annual review of biochemistry* **58**, 765-792 (1989).

18 Soskine, M. & Tawfik, D. S. Mutational effects and the evolution of new protein functions. *Nature Reviews Genetics* **11**, 572-582 (2010).

19 Guerois, R., Nielsen, J. E. & Serrano, L. Predicting changes in the stability of proteins and protein complexes: a study of more than 1000 mutations. *Journal of molecular biology* **320**, 369-387 (2002).

20 Taverna, D. M. & Goldstein, R. A. Why are proteins so robust to site mutations? *Journal of molecular biology* **315**, 479-484 (2002).

21 Taverna, D. M. & Goldstein, R. A. Why are proteins marginally stable? *Proteins: Structure, Function, and Bioinformatics* **46**, 105-109 (2002).

22 Bloom, J. D., Raval, A. & Wilke, C. O. Thermodynamics of neutral protein evolution. *Genetics* **175**, 255-266 (2007).

23 Zeldovich, K. B., Chen, P. & Shakhnovich, E. I. Protein stability imposes limits on organism complexity and speed of molecular evolution. *Proceedings of the National Academy of Sciences* **104**, 16152-16157 (2007).

24 Serohijos, A. W. & Shakhnovich, E. I. Merging molecular mechanism and evolution: theory and computation at the interface of biophysics and evolutionary population genetics. *Current opinion in structural biology* **26**, 84-91 (2014).

25 Bava, K. A., Gromiha, M. M., Uedaira, H., Kitajima, K. & Sarai, A. ProTherm, version 4.0: thermodynamic database for proteins and mutants. *Nucleic acids research* **32**, D120-D121 (2004).

26 Dasmeh, P. & Serohijos, A. Estimating The Contribution Of Folding Stability To Non-Specific Epistasis In Protein Evolution. *bioRxiv*, doi:10.1101/122259 (2017).

27 Geiler-Samerotte, K. A. *et al.* Misfolded proteins impose a dosage-dependent fitness cost and trigger a cytosolic unfolded protein response in yeast. *Proceedings of the National Academy of Sciences* **108**, 680-685 (2011).

28 Serohijos, A. W., Rimas, Z. & Shakhnovich, E. I. Protein biophysics explains why highly abundant proteins evolve slowly. *Cell reports* **2**, 249-256 (2012).

29 Ghosh, K. & Dill, K. Cellular proteomes have broad distributions of protein stability. *Biophysical journal* **99**, 3996-4002 (2010).
